# The complete chloroplast genome sequence of *Ficus beipeiensis* (Moraceae), an endemic and endangered plant in China

**DOI:** 10.1080/23802359.2020.1861556

**Published:** 2021-02-19

**Authors:** Fang Han, Jingling Li, Jing Yuan, Jie Yu

**Affiliations:** a College of Horticulture and Landscape Architecture, Southwest University, Chongqing, China; b Key Laboratory of Horticulture Science for Southern Mountainous Regions, Ministry of Education, Chongqing, China

**Keywords:** *Ficus beipeiensis*, chloroplast genome, phylogenetic analysis, endangered, endemic plant

## Abstract

*Ficus beipeiensis* S.S.Chang is an evergreen plant of *Ficus* from the family Moraceae. This is an endangered species endemic to China. In terms of economic value, *F. beipeiensis* are used as a native plant resource of urban landscaping in Chongqing, China. Here, we sequenced, assembled and annotated the chloroplast (cp) genome of *F. beipeiensis*, and aim to provide genetic resources for this endangered species. The length of cp genome was 160,595 bp, with a large single-copy region (LSC) of 88,683 bp and a small single-copy region (SSC) of 20,160 bp separated by a pair of inverted repeats (IRs) of 25,876 bp. It encodes 110 unique genes, including 76 protein-coding genes, 30 transfer RNA genes, and 4 ribosomal RNA genes. Besides, we reconstructed the phylogeny of Moraceae based on the whole cp genome sequences data set. Phylogenetic analysis shows that all analyzed *Ficus* species are clustered and form a monophyletic group. *Antiaris* is a sister group to *Ficus*. In our maximum likelihood (ML) tree, *F. beipeiensis* is closely related to *F. racemose*.

*Ficus beipeiensis* S.S.Chang is an evergreen plant of *Ficus* genus in the family Moraceae, which is endemic to China (Wu et al. 2003). This species grows at altitudes of 300–500 meters and often grows in damp places above or below steep limestone. In terms of economic value, *F. beipeiensis* are used as a native plant resource of urban landscaping in Chongqing, China, which has excellent carbon fixation and dust retention ability (Shi et al. 2018). However, the survival status of wild species of *F. beipeiensis* are extremely dangerous, and it was recorded in China Species Red List in 2004. Unfortunately, there is little research focused on its genetic information, which limits our further understanding of this species. In this study, we sequenced, assembled and annotated the cp genome of *F. beipeiensis*, and aim to provide genetic resources for this endangered species. The results obtained here will greatly contribute to the conservation of this endemic and endangered species. Besides, it is of great value to the classification and evolutionary history of *Ficus*.

The fresh leaves of *F. beipeiensis* were collected from North Hot Spring, Beibei District, Chongqing (Geospatial coordinates: N29.83003, E106.426292), and the samples were deposited in the Herbarium of Southwest University, Chongqing. We used the genomic DNA kit (Tiangen Biotech, Beijing) to extract the total DNA; the purity and integrity of DNA were determined by agarose gel electrophoresis. The DNA library was constructed with 1μg DNA using the library preparation kit (New England BioLabs, America), and sequenced by using the Illumina NovaSeq 6000 sequencing platform. A total of 6.39 G raw data were generated. Totally 21,111,373 clean reads were obtained by removing low-quality sequences: sequences with a quality value of Q≦5 accounted for more than 50% of the total base, and sequences with more than 10% bases being ‘N’. The *de novo* genome assembly from the clean data was accomplished utilizing the NOVOPlasty (v.2.7.2) (Dierckxsens et al. 2017). We annotated the genome by using CPGAVAS2 (Shi et al. 2019), and manually edited problematic annotations using Apollo (Misra and Harris 2006). The complete cp genome sequence has been deposited in GenBank with accession number MT611420 (https://www.ncbi.nlm.nih.gov/nuccore/MT611420).

The cp genomes of *F. beipeiensis* are characterized by a typical circular DNA molecule with a length of 160,595 bp. It has a conservative quartile structure consisting of a large single-copy (LSC) region, a small single-copy (SSC) region, and a pair of inverted repeat (IRs) regions, with lengths of 88,683 bp, 20,160 bp, and 25,876 bp, respectively. The GC content analysis showed that the overall GC content of the total length, LSC, SSC, and IR regions were 35.87%, 33.52%, 28.87%, and 42.63%, respectively, which was similar to other taxa in *Ficus* (Mao and Bi 2015). Besides, a total of 128 genes are annotated, and 110 are unique genes, including 76 protein-coding genes, 30 tRNA genes and 4 rRNA genes, respectively.

Furthermore, we constructed the maximum likelihood (ML) tree by using the complete chloroplast genome sequences as the data sets. The cp genome sequences of 21 species were downloaded from GenBank. All cp genome sequences were aligned using MAFFT (v 7.450) (Rozewicki et al. 2019). The phylogenetic tree was constructed using the Maximum Likelihood (ML) method implemented in RaxML (v8.2.4) (Stamatakis 2014). The bootstrap analysis was performed with 1000 replicates. Our phylogenetic tree is divided into two subclades, corresponding to Moroideae and Antiaridoideae respectively (Figure 1). In this phylogenetic tree, the species, *F. beipeiensis* is closely related to *F. racemose*, and all 6 analyzed *Ficus* species are clustered and form a monophyletic group. Besides, *Antiaris* is a sister group to *Ficus*. This topology is consistent with the results of previous studies (Wang et al. 2019).

**Figure 1.**
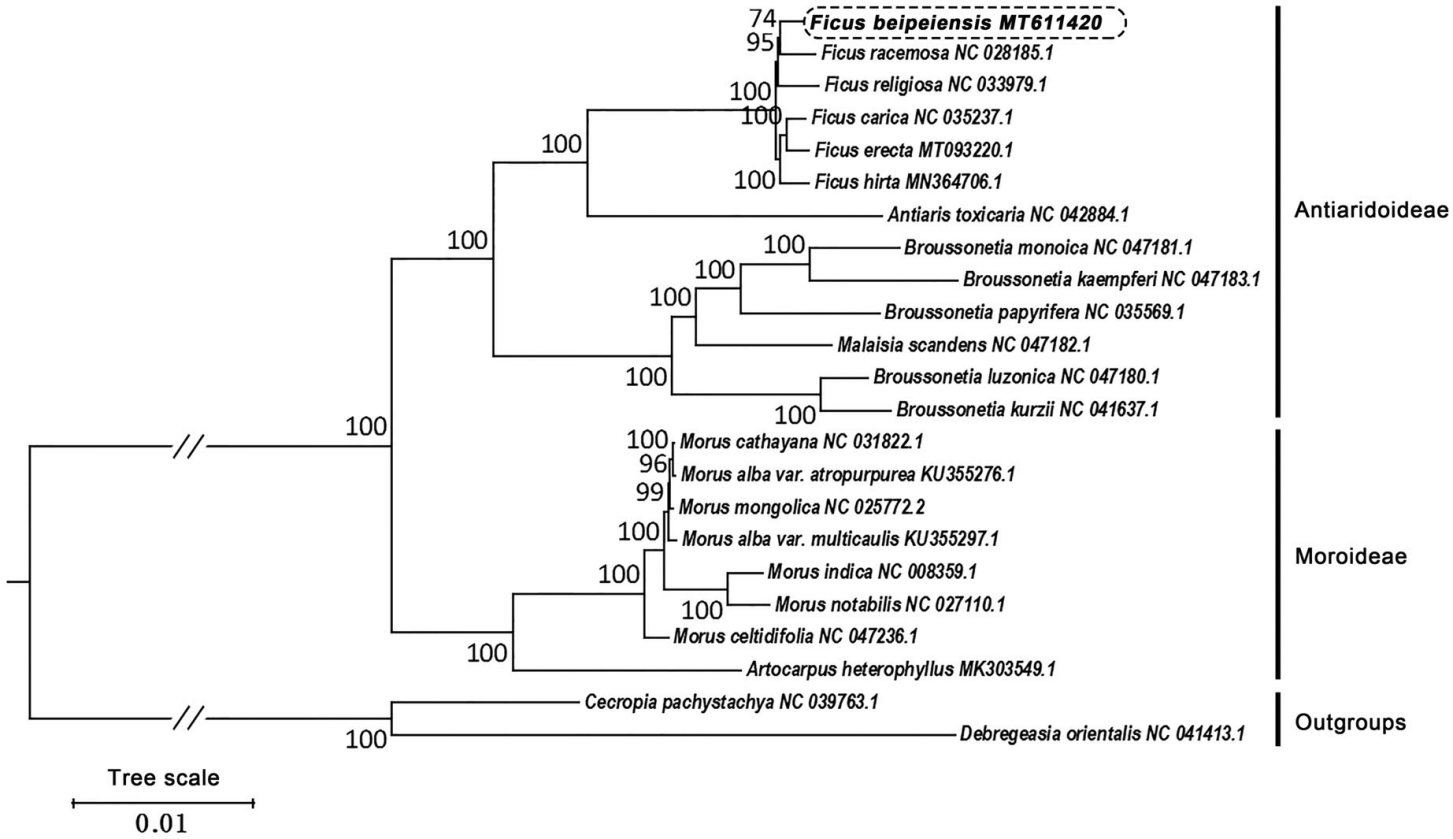
Phylogenetic relationships of 20 species from Moraceae inferred using the Maximum likelihood (ML) method. The phylogenetic tree constructed using the complete chloroplast genome sequences among the 20 species. Numbers near the nodes represent ML bootstrap values. Relative branch lengths are indicated. Bootstrap values were calculated from 1000 replicates. Two taxa, namely, *Cecropia pachystachya* and *Debregeasia orientalis* were used as outgroups.

## Data Availability

The authors confirm that the data supporting the findings of this study are available. The genome sequence has been deposited in GenBank with accession numbers MT611420 (https://www.ncbi.nlm.nih.gov/nuccore/MT611420). The sample has been deposited in the Herbarium of Southwest University in Chongqing, China with the accession number: SWU-BBR0413-01.
